# Electric Field-Responsive Mesoporous Suspensions: A Review

**DOI:** 10.3390/nano5042249

**Published:** 2015-12-15

**Authors:** Seung Hyuk Kwon, Shang Hao Piao, Hyoung Jin Choi

**Affiliations:** Department of Polymer Science and Engineering, Inha University, Incheon 402-751, Korea; E-Mails: 22141016@inha.edu (S.H.K.); sanghoo1105@inha.edu (S.H.P.)

**Keywords:** electrorheology, mesoporous, suspension, nanocomposite

## Abstract

This paper briefly reviews the fabrication and electrorheological (ER) characteristics of mesoporous materials and their nanocomposites with conducting polymers under an applied electric field when dispersed in an insulating liquid. Smart fluids of electrically-polarizable particles exhibit a reversible and tunable phase transition from a liquid-like to solid-like state in response to an external electric field of various strengths, and have potential applications in a variety of active control systems. The ER properties of these mesoporous suspensions are explained further according to their dielectric spectra in terms of the flow curve, dynamic moduli, and yield stress.

## 1. Introduction

**Figure 1:**
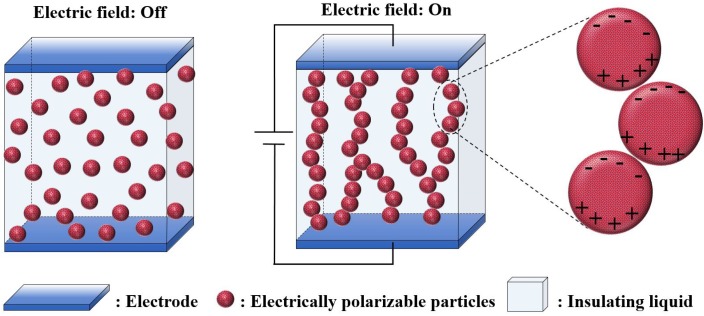
Schematic diagram of the electrorheological (ER) phenomenon under an applied electric field.

“Smart” materials that can respond to external stimuli, such as temperature, pH, light, mechanical stress, and electric/or magnetic fields, have attracted considerable attention in both academia and industry [1,2,3,4]. Among these stimuli-responsive smart materials, electro-responsive electrorheological (ER) particles are a class of smart or intelligent materials. ER suspensions generally consist of electrically-polarizable particles dispersed in an insulting liquid, such as silicone oil or mineral oil, demonstrating electrically-tunable viscoelasticity [5,6,7,8,9]. As shown in Figure 1, electrically-polarizable particles dispersed in an insulating medium can be polarized instantaneously under an electric field, in which the particles tend to aggregate and form a columnar structure along the applied electric field direction due to the induced dipole-dipole interaction. Therefore, their rheological properties, such shear stress, shear viscosity, yield stress, dynamic moduli, and stress relaxation, can be altered rapidly from liquid-like to solid-like by applying an external electric field. Many ER fluids are known to follow a Bingham fluid model with non-Newtonian fluid behavior. The characteristics of this model suggest that the fluid has yield stress and its flow movement is prevented when the applied shear stress is less than its yield stress [10]. With its yield stress, its controllable and tunable phase change has huge industrial applications [8] in many areas, such as dampers [11], clutches [12], shock absorber systems, microfluidics [13,14,15], ER polishing [16], ER haptic device [17], and tactile displays [18].

A range of materials have been used as electrically-polarizable particles in ER fluids, including organic and inorganic particles, which can be classified as either an intrinsically polarizable anhydrous or extrinsically polarizable hydrous system [19]. Earlier studies of ER fluids focused on extrinsically-polarizable particles, such as silica [20], alumina, and starch [21,22], as well as cellulose with various additives, including water and surfactants to generate polarizability [23,24]. On the other hand, these particles generally have drawbacks in their applications, including device corrosion and low thermal stability due to such additives. To overcome these drawbacks, a range of intrinsically-polarizable anhydrous ER materials have been developed. These materials, which contain semi-conducting polymer particles, such as polyaniline (PANI) [25,26,27], copolyaniline (COPANI) [28,29], polypyrrole (PPy) [30,31], poly(p-phenylene) [32,33], and carbonized materials [34,35], are polarized in an electric field due to electrons within the polymer molecule.

Mesoporous materials possessing a uniform mesopore structure with pore sizes in the range, 2–50 nm, and extremely high specific surface areas, have been studied extensively [36] for their potential applications as catalysts [37], separation, optical materials [38], and nanotechnology since the first report of the synthesis of MCM-41 (Mobil Composition of Matter No. 41) [39]. These materials have significantly larger pores than conventional molecular sieves and zeolites. Another related family of mesoporous compounds was introduced with the preparation of mesoporous silica by the layered poly(silicate kanemite) (NaHSi_2_O_5_·3H_2_O) [40]. The disclosure by Mobil of the M41S family of materials possessing large uniform pore structures, high specific surface areas, and specific pore volumes depending on the channel arrangement, was an important breakthrough in mesoporous materials research [39]. Although all three phases in the M41S family can be synthesized by varying the reaction conditions slightly, the energetic stability decreases in the order of hexagonal MCM-41 [41,42], cubic MCM-48 [43] and lamellar phase MCM-50 [44]. Among the M41S families, MCM-41 type materials have been studied most frequently [45]. They consist of uniform, hexagonal arrays of mesoporous with pore openings between 2 and 10 nm, and offer unique opportunities for the preparation of new functional composite materials [46]. Recently, the ER behavior induced by MCM-41-based suspensions has been reported [47]. Further studies led to the discovery of several other types of mesoporous silica with narrow pore-size distributions and well-defined structures. Stucky and coworkers [48] proposed the new synthesis of ordered hexagonal mesoporous silica, called SBA-15 using triblock poly(ethylene oxide)-poly(propylene oxide)-poly(ethylene oxide) copolymers as the structure-directing agents in an acidic medium. The most notable member of this family of materials is hexagonally-ordered SBA-15 mesoporous silica, which has pore sizes in the range 3–10 nm, depending on the synthetic conditions.

On the other hand, zeolites, microporous aluminosilicate minerals, have been also applied as ER materials because of their easy accessibility and high ER effect. The principal synthetic zeolites in commercial use include Linde Type-A (LTA) and Linde Type-X. For ER applications, different types of zeolites, such as 3A, 4A, 5A, 13X, and even Y-Type have been investigated [49,50], showing different ER effects owing to their different structural components and pore size in their frameworks. Tian *et al.* [51] synthesized a zeolite Y-based ER fluid with high shear yield stress (>20 kPa) in a 5 kV/mm electric field by introducing a post-microwave treatment.

This paper briefly reviews various mesoporous materials and their conducting nanocomposites employed in electro-responsive ER fluids, including the synthesis method, morphological characteristics and typical physical characteristics. In addition, the ER effects in dispersions of mesostructured (filled with conducting polymers) materials were compared based on their rheological characteristics, such as flow curve, shear viscosity, yield stress, and dynamic moduli, under an applied electric field of different strengths. This approach allows an examination of the contribution of polarization of a dispersed phase material formed from a porous dielectric matrix in mesoporous materials or those filled with molecules possessing certain conductivity in conducting mesoporous nanocomposites associated with conducting polymers related to the ER effect.

## 2. Fabrication of Electro-Responsive Mesoporous Materials

MCM-41 is generally synthesized using an aqueous solution of hexadecyl trimethyl ammonium chloride (HTACl), NH_3_ solution, and sodium silicate. Pure silica MCM-41 was obtained through a hydrothermal procedure using salts to improve the hydrothermal stability in addition to pH adjustments during the hydrothermal process for the structural order [52]. Pure MCM-41 particles have been used as ER materials. On the other hand, they exhibit a lower ER effect compared to traditional inorganic materials when adopted directly as an ER dispersant. To overcome the disadvantages of MCM-41 materials, conducting polymers were introduced to the mesopores to prepare conducting polymer/MCM-41 composites, and a range of conducting polymer/mesoporous material composites were reported to show stronger ER properties compared to the original mesoporous material [8].

**Figure 2:**
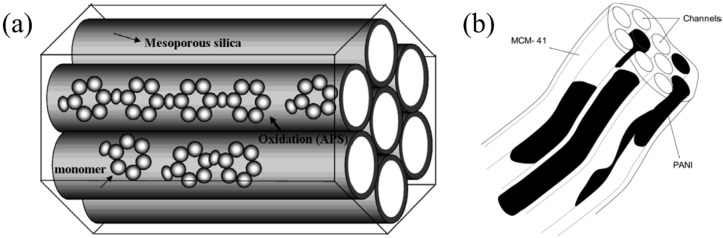
(**a**) Proposed schematic diagram of conducting polymer/Mobil Composition of Matter No. 41 (MCM-41) nanocomposite particles (Reproduced with permission from [58]. Copyright Elsevier, 2009); (**b**) in which the conducting polymer can be polyaniline (Reproduced with permission from [56]. Copyright American Chemical Society, 2004).

Zhu *et al.* [53] synthesized copper phthalocyanine (CuPC)-doped MCM-41 via the *in situ* micelle-assisted incorporation of CuPC during MCM-41 synthesis, according to the suggested modified method [54], where a low pH condition was replaced with a high pH condition. Cheng *et al.* [55] reported modified MCM-41 by triethanolamine (TEA) through a wet impregnation method. In principle, TEA is deposited on the inner walls of the MCM-41 channels, expecting hydrogen bonding between TEA and MCM-41. Conducting PANI/MCM-41 was also reported [56]. To prepare the PANI/MCM-41 nanocomposite, MCM-41 containing aniline was immersed in a HCl aqueous solution, and the oxidant initiator was added to the reaction system with continuous stirring at room temperature. PANI was synthesized through chemical oxidation polymerization. Furthermore, to examine the effects of the modified structure of copolyaniline (COPANI) in mesoporous nanocomposites on the ER performance compared to pure PANI, a COPANI-based composite polymerized from methylaniline with a methyl group on its main chain was fabricated using the same method for synthesizing PANI/MCM-41 [57]. The COPANI/MCM-41 based ER fluid possessed relatively lower yield stress compared to that of the PANI/MCM-41-based ER fluid because of the lower conductivity of the COPANI than that of PANI. Figure 2a presents a suggested schematic diagram of typical conducting polymers within the MCM-41 channels [58]. A conducting polymer/MCM-41 nanocomposite material can be electrically anisotropic because of the conducting polymer forming predominantly inside the channels, as illustrated in Figure 2b. In addition, although conducting polymers cannot be used directly as ER materials without a de-doping process because they possess higher electrical conductivity immediately after polymerization, such a dedoping process is unnecessary when using mesoporous materials because they play the role of an insulating material in nanocomposites [57].

Fang *et al.* [59,60] reported another conducting polymer of PPy-modified specific MCM-41 composites. Compared to the conventional method of synthesizing MCM-41, in which aqueous colloidal silica in a tetraethyl ammonium hydroxide solution was combined with a cethyltrimethyl ammonium chloride solution, the swollen MCM-41 with a large pore diameter was prepared [61]. Here, PPy was synthesized by chemical oxidation polymerization, such as PANI. Cho *et al.* [62] reported the preparation of mesoporous SBA-15 based nanocomposite, in which aniline was inserted and then polymerized inside the channels of SBA-15. On the other hand, for the grafting of PANI moieties through *in situ* polymerization into mesoporous silica SBA-15, Sasidharan *et al.* [63] adopted monolayer *N*-propylaniline-functionalized SBA-15, finding that the grafted PANI exhibits ER effects with an increased shear viscosity under an applied electric field compared with the non-grafted PANI nanocomposites.

Concurrently, with regard to zeolite-based ER fluid research, Cho *et al.* [64] examined the rheological and dielectric properties of ER fluids prepared from commercial zeolites, 3A, 5A, and 13X suspended in silicone oil. A study of the rheological properties of ER fluids using zeolite with liquid crystal (LC) additives [65] revealed the LC component in the ER fluid to increase the yield stress by the synergistic effect of the orientation of LC under an applied electric field.

In addition, a combination of the sol-gel process and self-assembly under hydrothermal conditions has been adopted widely as the synthetic method towards a range of mesoporous silica materials. In this process, silica precursors are dissolved in an aqueous solution containing a surfactant. The silica precursors then undergo hydrolysis and condensation to form a solution called a sol, which contain oligomeric and polymeric silicate species. In the presence of a surfactant and their aggregates, the hydrolysis and condensation reactions of silica precursors lead to the formation of organic-inorganic species that become increasingly polymerized and form a gel as the reactions proceeds. The self-assembled surfactant-silicate material ultimately precipitates from the solution. Recently, the ER property of graphene [66] and graphene oxide [67]-modified mesoporous silica was reported. On the other hand, conducting polymer/mesoporous nanocomposites have been applied further to wastewater treatment by removing toxic heavy metal ions, such as mercury. Tang *et al.* [68] synthesized a poly(aniline-co-*o*-aminophenol) (PAOA)/mesoporous silica SBA-15 nanocomposite and examined its aqueous Hg^2+^ removal properties. They reported that while the SBA-15 template has a remarkably large surface area for coating PAOA, PAOA provides plentiful amidocyanogen as the major binding sites for mercury removal with intrinsic low dependence on the pH. PANI/hexagonal mesoporous silica nanocomposites [69] were also applied to waste water treatment. Furthermore, a conducting polypyrrole/SBA-15 nanocomposite was applied for the molecular imprinting of ascorbic acid recognition [70]. The mesostructures of the mesoporous nanocomposites produced by confined synthesis were reported to be useful templates for the fabrication of highly-ordered mesostructured nanowires and nanowire arrays [71].

## 3. Characterization

### 3.1. Electron Microscopy

**Figure 3:**
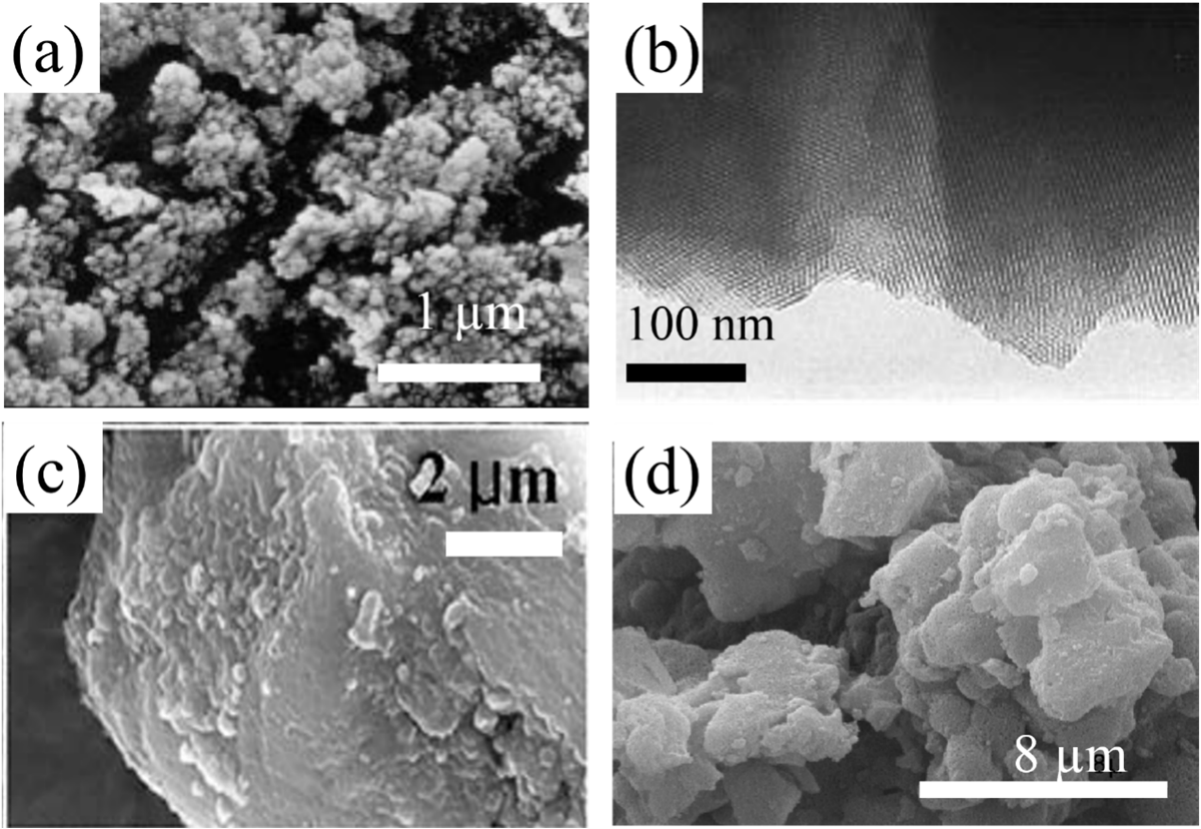
(**a**) Scanning electron microscopy (SEM) and (**b**) transmission electron microscopy (TEM) image of MCM-41 particles (Reproduced with permission from [47]. Copyright Elsevier, 2000); (**c**) SEM images of copolyaniline (COPANI)/MCM-41 particles (Reproduced with permission from [58]. Copyright Elsevier, 2009); (**d**) PPy/MCM-41 particles (Reproduced with permission from [60]. Copyright Elsevier, 2008).

The morphology of the pure MCM-41 particles and conducting polymer-modified MCM-41 composites were examined by both scanning electron microscopy (SEM) and transmission electron microscopy (TEM). Figure 3a shows a SEM image of pure MCM-41 particles, demonstrating different shapes of particles with a wide range of particle sizes from less than 300 nm to more than 5 µm [47]. The morphology and microstructure of the MCM-41 samples obtained were examined by transmission electron microscopy (TEM). Figure 3b presents the uniform hexagonal shaped mesopore structure of MCM-41 particles. According to TEM, the MCM-41 crystals prepared were comprised mostly of particles, 80–120 nm in size, and existed as agglomerated groups. Figure 3c,d presents SEM images of COPANI/MCM-41 and PPy/MCM-41 nanocomposite particles, respectively. Although some aggregates of MCM-41 particles were visible, the size of the primary particles was less than 10 µm. No apparent difference in the particle surface morphology between the pristine MCM-41 and polymer-coated MCM-41 was observed. This could be considered indirect evidence that the conducting filaments were prepared within the nano-scaled hexagonal channels of the MCM-41 rather than surface adsorption, which led to subsequent growth of the conducting polymer chains. The conducting polymer product confined within the MCM-41 channels could also be confirmed by nitrogen adsorption-desorption isotherm analysis.

### 3.2. X-Ray Diffraction

**Figure 4:**
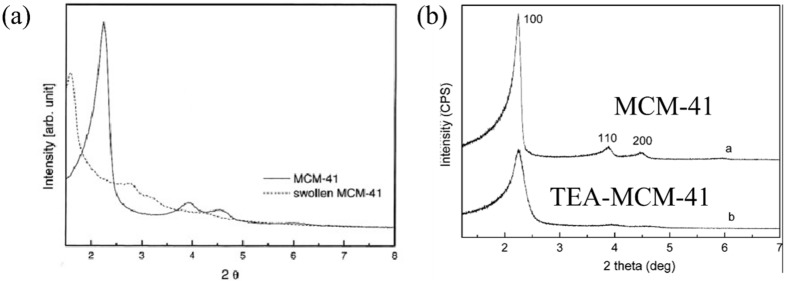
(**a**) X-Ray diffraction (XRD) patterns of pure and swollen MCM-41 (Reproduced with permission from [57]. Copyright Elsevier, 2005); (**b**) polymer modification MCM-41 particles (Reproduced with permission from [55]. Copyright Elsevier, 2008).

Figure 4 presents the X-ray diffraction (XRD) patterns of the as-prepared MCM-41 and polymer-coated MCM-41 materials, respectively, showing the reflection peaks in the low angle region, characteristic of mesopore structures. The XRD pattern corresponds to regular hexagonal channels of a pure MCM-41structure with a strong (100) peak followed by (110) (200) and (210) peaks in the 2°–7° 2θ range, as shown in Figure 4a. The peaks were indexed to a hexagonal lattice typical for MCM-41 materials [39]. The peak of swollen MCM-41 was shifted to a smaller angle due to the increased pore size compared to that of MCM-41.

After modification with the polymer, a significant decrease in peak intensities occurred upon polymerization, as shown in Figure 4b, which was attributed to the filling of pores with a polymer. This has been observed in many systems, in which the polymer filaments were synthesized within the channels of the mesoporous materials [72].

### 3.3. BET Analysis

**Figure 5:**
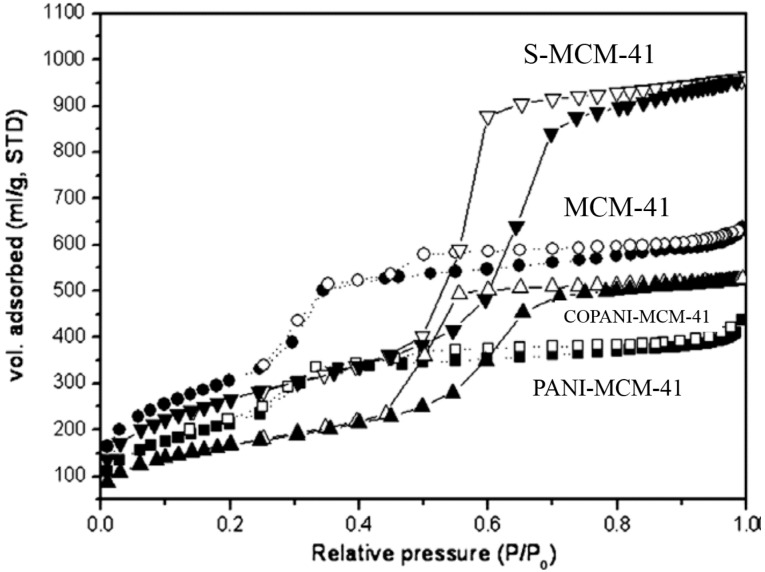
N_2_ adsorption-desorption isotherm plots before polymerization, MCM-41 (●) and swollen MCM-41 (▼); after polymerization, COPANI/swollen MCM-41 (▲) and PANI/MCM-41 (■), respectively (Reproduced with permission from [58]. Copyright Elsevier, 2009).

Adsorption-desorption isotherm analysis was carried out to estimate the specific surface area and pore properties of the mesoporous particles. The N_2_ adsorption-desorption isotherm is an effective way of confirming the successful location of the polymers within the channels. Figure 5 shows the N_2_ adsorption-desorption isotherm plots of pure MCM-41 and swollen MCM-41 particles, as well as the composites with a polymerized conducting fiber within the channels, as pointed with different symbols. According to the sorption analysis data, the pore volume of PANI/MCM-41 was reduced compared to the pure MCM-41 [58]. In the case of COPANI/swollen MCM-41, the residual pore volume was also reduced compared to the swollen MCM-41.

## 4. Electrorheological Characteristics

### 4.1. Flow Curve Test

The rheological characteristics of ER fluids have been the main focus for several decades. Many review papers covered not only different ER materials, but also their rheological understanding [73,74]. Here, the ER properties of MCM-41 and conducting polymer-coated MCM-41 particles were examined using a rotational rheometer equipped with a DC high voltage generator. The measuring geometry consisted of a concentric cylinder geometry. Cho and his group [56,62] mainly used a Couette-type rotational rheometer (Physica, Anton-Paar, Graz, Austria), in which the concentric cylinder measuring systems require relatively large sample volumes and are more difficult to clean. On the other hand, they could work with low shear viscosity materials and mobile suspensions, such as ER fluids. Their large surface area gives them a greater sensitivity, so they can produce good data in a low shear rate test. Therefore, the concentric cylinder geometry is suitable for examining the ER fluid flow behavior due to the near constant shear rate across the gap [75,76]. A rotational rheometer with two parallel plate systems has been also used for the ER measurement of mesoporous cerium-doped titania particles synthesized using a block-copolymer template [77]. Regarding the PANI/MCM-41- and COPANI/MCM-41-based ER fluids, Fang *et al.* [58] reported that the PANI/MCM-41-based ER fluid had higher shear stress than PANI system at both the high and low shear rate regions. Nevertheless, the COPANI/S-MCM-41-based ER fluid generated a relatively lower yield stress than the PANI/MCM-41-based ER fluid because of the lower conductivity of the COPANI than that of PANI.

On the other hand, a typical rotational test can be set up to observe the flow curve with a controlled shear rate (CSR) mode or controlled shear stress (CSS) mode. The flow curves of the ER fluids obtained from CSR mode indicated an increase in shear stress (τ) and shear viscosity (η) with an increasing shear rate. Moreover, the dynamic yield stress can be obtained by extrapolating the shear stress at a zero shear rate limit from the flow curve. On the other hand, the CSS mode can be used to obtain a static yield stress. These are useful for observing both the dynamic and static yield stresses of ER fluids, as well as their dependence on the electric field strength [8].

**Figure 6:**
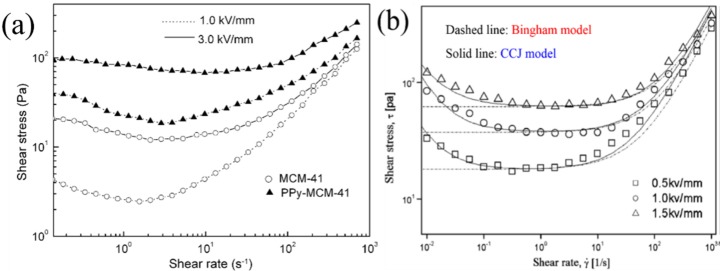
(**a**) Shear stress curve as a function of the shear rate for PPy/MCM-41 ER fluids (triangle) and MCM-41-based ER fluid (circle) under different applied electric fields. (Reproduced with permission from [78]. Copyright Elsevier, 2006); (**b**) Fitting curve of the model equations to shear stress curves for PPy/MCM-41 nanocomposite-based ER fluids under three different electric field strengths (Reproduced with permission from [59]. Copyright Elsevier, 2010).

To determine the effects of PPy in the channels of MCM-41, the shear stress *versus* shear rate dependence of pure MCM-4- and PPy-doped MCM-41 materials (10 wt % ER fluids) are plotted in Figure 6a at different electric field strengths [78]. The shear stress increased with increasing shear rate in the absence of an applied electric field. In the presence of an electric field, however, the shear stress increases with increasing electric field strength. These results suggest that conducting PPy encapsulated in the channels of MCM-41 is a more efficient polarization source in ER fluids than water.

Figure 6b presents that the shear stress curves of the PPy/MCM-41-based ER fluid, as a function of the shear rate for three electric field strengths, were fitted using both the conventional Bingham model (dashed lines) and Cho-Choi-Jhon (CCJ) model (solid lines). Initially, the simple Bingham model was used by introducing the dynamic yield stress (τ*_y_*) and shear viscosity (η) as follows:

(1) $$\begin{array}{l} \tau =\tau_{y}+\eta_{0}\dot{\gamma },\tau \geq \tau_{y}\\ \dot{\gamma }=0,\tau <\tau_{y} \end{array}$$

where τ is the shear stress, τ_0_ is the yield stress, which is related to the electric field, γ̇ is the shear rate, and η_0_ is the shear viscosity. On the other hand, the shear stress behavior as a function of the shear rate applied in Figure 6a is quite different from a classical Bingham fluid. The shear stresses that decreased slightly or remained constant with increasing shear rate at certain shear rate zones were widened as the electric field was increased. Therefore, a rheological equation of state called the CCJ model was introduced to fit the shear stress curve for a wide range of ER fluids, particularly in the low shear rate region [8,75,79]. The CCJ model has been used to analyze the shear stress characteristics by fitting the flow curves with six parameters, as represented using the following equation [28]:

(2) $$\tau =\;\frac{\tau_{y}}{1+{\left(t_{1}\dot{\gamma }\right)}^{\alpha }}+\eta_{\infty }\left(1+\frac{1}{{\left(t_{2}\dot{\gamma }\right)}^{\beta }}\right)\dot{\gamma }$$

While the first term in the right hand side of Equation (2) implies shear stress behavior at a low shear rate region with the decrease of shear rate, the second one controls the curve fitting in the high shear rate region, where the shear stress increases with the shear rate. Here, τ*_y_* is the dynamic yield stress defined as the extrapolated shear stress from the low shear rate region [80], and η_∞_ is the shear viscosity at an infinite shear rate. *t*_1_ and *t*_2_ are time constants. The parameters, α and β, are in charge of the decrease and increase in shear stress, respectively, in the low and high shear rate regions. The exponent, β, has the range, 0 < β ≤ 1, because dτ/dγ̇ ≥ 0 above the critical shear rate [81]. The CCJ model has been also adopted in various ER and magnetorheological (MR) materials [82].

The complicated behavior of the ER fluids better corresponded to the CCJ model than the other fluid model [8,67], and the decrease in shear stress was explained from the mechanism that in a low shear rate region, the electrostatic interactions among dispersed particles induced by external electric fields are dominant compared to the hydrodynamic interactions induced by the external flow field [58] investigated using a rotational rheometer. The initially-aligned chain-like structures begin to break due to shear deformation, while the broken structures reform the chains under an applied electric field strength, depending on the magnitude of the applied shear and particle-particle interaction in the fibrils [8]. A decrease in shear stress was observed when the increase in the re-formed structures with shear rate is not as complete as those before [8] applying shear flow, suggesting that as the shear rate increases, the rate of fibril destruction becomes faster than the formation rate [79,81]. This proposed mechanism has been also interpreted from the dielectric spectra [83]. Recently, an improved rheological equation of state to explain these shear stress behaviors was further introduced with fewer parameters. Seo and Seo [84] proposed a four-parameter model to explain the structural deformations associated with yielding at different stress levels of ER fluids.

On the other hand, for polymer solutions and colloidal dispersions, similar behavior of the shear stress decreased as a function of the shear rate below a critical shear rate, which has been reported widely as shear banding phenomena [85], in which the system becomes mechanically unstable in a regime where the shear stress decreases with increasing shear rate. Typically, the fluid separates in a low and high shear band, resulting in a shear stress does not vary with a shear rate in this regime. Even in the cases of ER fluids, several groups report such a phenomenon of shear banding. Volkova *et al.* [86] reported shear banded flows in ER and MR fluids, in which a sudden jump of shear stress and the start of a layered stripe pattern are observed. They explained this phenomenon based on the phase transition from a nematic-like phase to an isotropic state under the applied external field which is obtained when the shearing hydrodynamic breaking forces on a pair of particles overcome either electrostatic or magnetic forces in the case of ER or MR fluids, respectively [86]. A two-fluid continuum model was also introduced to explain mass transport in ER fluids [87], and by solving mass balance, stripe formation related to shear banding is found to be deduced when an ER fluid is subjected simultaneously to an applied electric field and shear flow, while the column is being formed in the absence of flow. Such a shear band has been also observed for giant ER fluids [88,89].

Concurrently, the correlation between the yield stress and electric field can be expressed as:

(3) $$\tau_{y}\propto E^{m}$$

Regarding the origin of this relationship, the applied electric field induces electrostatic, polarized interactions among the dispersed particles and also between the dispersed particles and electrodes [8]. Therefore, the solid-like characteristics of the yield stress, which can be modeled by the power-law equation of the applied electric field strength, mainly follows the polarization model with a slope of 2.0, where the dielectric constant mismatch between the particles and medium oil is crucial for the attractive inter-particle force [90].

On the other hand, the polarization model does not describe the flow effect accurately for either the high electric field applied or for high particle volume concentration. In that case, the ER performance is affected by the conductivity mismatch and the interaction between the particles and the medium oil [91].

Various ER fluids show different exponents with Equation (3) from either 1.5 for the conduction model [92] or 2.0 for the polarization model [93]. Note that a polar molecule-dominated mechanism was also proposed for an extremely high ER effect with giant ER fluids induced by modified dielectric nanoparticles [7].

**Figure 7:**
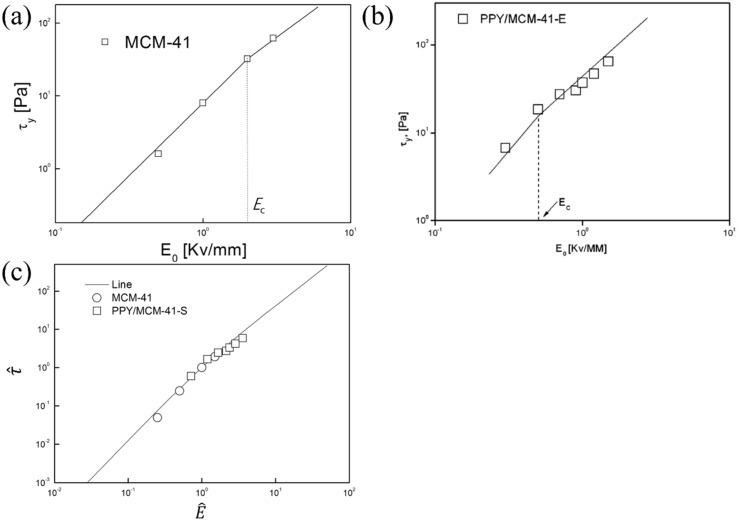
(**a**) Dynamic yield stress of MCM-41-based ER fluids at four different electric field strengths; (**b**) Dynamic yield stress of PPy/MCM-41-based ER fluids at seven different electric field strengths (Reproduced with permission from [59]. Copyright Elsevier, 2010); (**c**) $\hat{\tau }$ *vs.* $\hat{E}$ for pure MCM-41 and PPy/MCM-41-based ER fluids. The solid line is drawn with Equation (5).

Figure 7a,b shows the dynamic yield stresses of two different ER fluids based on pure MCM-41 [47] and PPy-MCM-41 particles, respectively, as a function of the electric field strength, in which the dynamic yield stress implies the shear stress at a zero shear rate limit from their corresponding flow curves. As shown in Figure 7a,b, the critical electrical field strength (*E_c_*) was estimated to be 2 and 0.5 kV/mm for pure MCM-41 and PPy-MCM-41, respectively.

The dynamic yield stress data of the MCM-41 based ER fluids are plotted in Figure 7a, and are correlated with the universal scaling relationship between the electric field strength (*E*_0_) and dynamic yield stress via Equation (4) suggested by Choi *et al.* [94]:

(4) $$\tau_{y}\left(E_{0}\right)=\alpha {E_{0}}^{2}\left(\frac{\tan h\sqrt{E_{0}/E_{c}}}{\sqrt{E_{0}/E_{c}}}\right)$$

where α depends on the dielectric constant of the fluid and the particle volume fraction, and *E_c_* is the critical electric field strength, which is related to the particle conductivity and concentration. Equation (4) suggests that τ*_y_* is proportional to *E*^2^ for *E*_0_ << *E_c_*, while switching abruptly to *E*^1.5^ for *E*_0_ >> *E_c_*. Equation (5) was derived using a generalized scaling function:

(5) $$\hat{\tau }=1.313{\hat{E}}^{3/2}\tan h\sqrt{\hat{E}}$$

The rearranged data in Figure 7a,b was fitted to a single line using Equation (5), as shown in Figure 7c. All new points for each ER fluid are in a universal line, indicating the significance of Equation (5) in constructing the yield stress master curve for ER fluids by arranging the experimental data with the corrected parameters. According to Figure 7c, the data obtained from Figure 7a,b collapsed onto to a single line using Equation (5). Therefore, this universal yield stress equation is believed to be very useful for constructing the master curve for ER fluids. Moreover, Equation (5) has been applied successfully to the MR fluid systems under an applied magnetic field. On the other hand, Seo [95] recently proposed a modified model that agrees well with the original bi-power model under a high electric field strength.

### 4.2. Oscillatory Analysis

An oscillatory test is generally used to examine the viscoelastic behavior and the process of chain formation for ER fluids accurately. In a typical amplitude sweep, the changes in dynamic moduli are measured by increasing the applied strain. Owing to the advisably chosen optimal critical strain, the frequency sweep characteristics of the dynamic moduli were examined as a function of frequency under different electric field strengths. The behaviors of the storage modulus (G′) and loss modulus (G″) as a function of frequency were examined, in which G′ and G″ represent the elasticity and viscous behavior, respectively.

**Figure 8:**
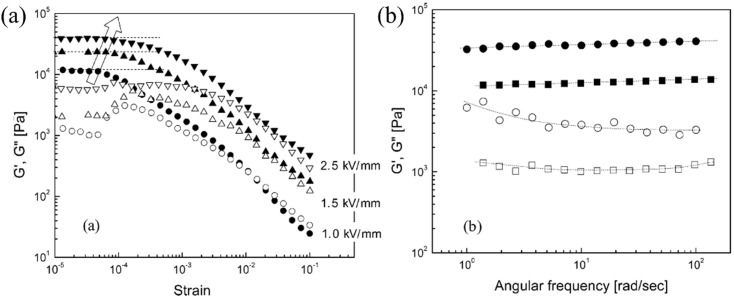
(**a**) Strain amplitude sweep of PANI/MCM-41 particles; (**b**) Angular frequency sweep of PANI/MCM-41 particle under 1 (■) and 2 (●) kV/mm using a strain of 3 × 10^−5^: Storage modulus (closed) and loss modulus (open) (Reproduced with permission from [56]. Copyright American Chemical Society, 2004).

Figure 8 shows the viscoelastic properties of the solidified PANI/MCM-41-based ER fluid under various electric fields. Figure 8a shows that G*'* is much higher than G″ under an applied electric field. This suggests that ER fluids are more solid-like rather than liquid-like under an applied electric field. A strain amplitude of 3 × 10^−5^ was selected in the plateau regime of G*'* and applied to perform the angular frequency sweep.

Figure 8b shows the results of the frequency sweep test from the oscillation measurements. Based on a properly chosen critical strain (3 × 10^−5^), the frequency sweep was examined under a range of electric field strengths. The G′ values were either constant or increased slightly with increasing deformation frequency up to 100 rad/s. The increase in G′ with increasing applied electric field indicates that the ER fluid becomes more elastic with the electric field under its linear viscoelastic conditions. Similar rheological and viscoelastic behaviors for their magnetically-analogous magnetorheological fluids under external magnetic fields have been also reported [96,97].

## 5. Dielectric Characteristics

**Figure 9:**
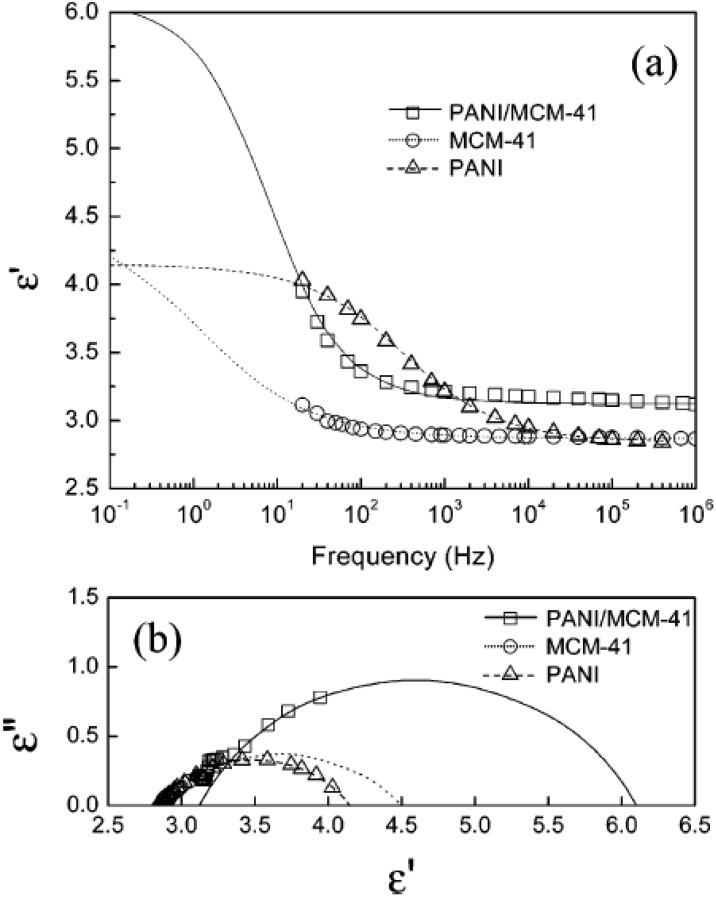
(**a**) Dielectric spectra (permittivity *versus* frequency); (**b**) Cole-Cole plot for each ER fluid (Reproduced with permission from [56]. Copyright American Chemical Society, 2004).

To examine the dielectric properties of ER fluids, the dielectric properties were measured using a LCR meter over the frequency range, 20–10^6^ Hz. Figure 9 shows the permittivity (ε′) as a function of frequency and Cole-Cole plots of the PANI/MCM-41-based ER fluid, respectively. The permittivity (ε′) depends on the polarizability and dielectric loss (ε″), describing the distribution of the relaxation times and stability of the interaction between particles. The lines in Figure 9 were fitted to the experimental data using a dielectric relaxation model, the Cole-Cole equation. The Cole-Cole equation is known to be helpful for obtaining the relationship between the dielectric properties and ER performances of ER fluids [98,99]. The model is described as follows:

(6) $$\varepsilon^{*}=\varepsilon^{\prime }+i\varepsilon^{\prime \prime }=\varepsilon_{\infty }+\frac{\varepsilon_{0}-\varepsilon_{\infty }}{1+{\left(i\omega \lambda \right)}^{1-\alpha }}\left(0\leq \;\alpha \;<1\right)$$

where ε^*^ is a complex dielectric constant, and ε′ and ε″ are the dielectric constant and dielectric loss, respectively. Δε is the difference between the dielectric constant when the frequency approaches zero and infinity (ε_0_ and ε_∞_). ω is the angular frequency. The exponent, 1 − α, characterizes the broadness of the relaxation time distribution. λ is the dielectric relaxation time, which is related to the rheological behaviors, such as yield stress under an applied electric field [83], where *f*_max_ is the maximum dielectric loss of an ER fluid. As a shorter λ within the adequate range and a larger Δε are applied, higher stress enhancement by the applied electric field is achieved, which are dependent on each other. λ can be estimated roughly from the relaxation of ε′ in Figure 9a, in which a reciprocal frequency at the maximum decreasing slope of ε′ is related linearly by λ. On the other hand, Δε can be obtained directly from the Cole-Cole plots (Figure 9b), in which Δε is the width of the Cole-Cole arc.

From the fitting results, the λ values for the MCM-41-, PANI/MCM-41-, and PANI-based ER fluids were 0.1, 0.02, and 0.0005 s, and the Δε values for the PANI-, MCM-41-, and PANI/MCM-41-based ER fluid were 1.31, 1.63, and 2.98, respectively. The conducting polymer coated-MCM-41 had a substantially larger Δε and shorter relaxation time, suggesting stronger attraction and a faster polarization rate of PPy-doped particles. Therefore, considering both λ and Δε, it can be concluded that the Δε value is more effective and dominant for the ER performance because it is related directly to the strength of the particulate fibril structures [100].

## 6. Conclusions

This review paper described some methodologies for the synthesis of smart mesoporous composite materials, such as MCM-41, conducting polymer/MCM-41 and conducting polymer/SBA-15 nanocomposites. The mesoporous composite were characterized by SEM, TEM, BET surface area measurements and XRD. The electric responsive mesoporous particles exhibited typical ER performance when dispersed in insulating oil, and are considered good candidates for electro-responsive ER materials with a large surface area. The shear stress curves were analyzed using the suggested CCJ model, which accurately fitted the unusual behavior in a low shear rate range compared to that fitted by Bingham model. Their yield stresses were analyzed quantitatively using the universal equation considering the dependence of the ER effect on the electric field strength. The dynamic properties of the mesoporous material-based ER fluid under various electric field strengths were also investigated at the critical strain, showing that the solid-like behavior of ER fluids increased with increasing applied electric field. These ER characteristics were also analyzed using the dielectric spectra of ER fluids.
